# Participation of Gα_i_-Adenylate Cyclase and ERK1/2 in Mas Receptor Signaling Pathways

**DOI:** 10.3389/fphar.2019.00146

**Published:** 2019-02-22

**Authors:** Valeria Burghi, Emiliana B. Echeverría, Máximo H. Sosa, Diego T. Quiroga, Marina C. Muñoz, Carlos Davio, Federico Monczor, Natalia C. Fernández, Fernando P. Dominici

**Affiliations:** ^1^Facultad de Farmacia y Bioquímica, Instituto de Investigaciones Farmacológicas (ININFA), Universidad de Buenos Aires, Consejo Nacional de Investigaciones Científicas y Técnicas, Buenos Aires, Argentina; ^2^Facultad de Farmacia y Bioquímica, Universidad de Buenos Aires, Consejo Nacional de Investigaciones Científicas y Técnicas, Instituto de Química y Fisicoquímica Biológicas (IQUIFIB), Buenos Aires, Argentina

**Keywords:** angiotensin-(1-7), cAMP, ERK, G protein-coupled receptor, Mas receptor, signaling

## Abstract

The MasR receptor (MasR) is an orphan G protein-coupled receptor proposed as a candidate for mediating the angiotensin (Ang)-converting enzyme 2-Ang-(1–7) protective axis of renin-angiotensin system. This receptor has been suggested to participate in several physiological processes including cardio- and reno-protection and regulation of the central nervous system function. Although the knowledge of the signaling mechanisms associated with MasR is essential for therapeutic purposes, these are still poorly understood. Accordingly, in the current study we aimed to characterize the signaling pathways triggered by the MasR. To do that, we measured cAMP and Ca^2+^ levels in both naïve and MasR transfected cells in basal conditions and upon incubation with putative MasR ligands. Besides, we evaluated activation of ERK1/2 by Ang-(1–7) in MasR transfected cells. Results indicated the existence of a high degree of MasR constitutive activity toward cAMP modulation. This effect was not mediated by the PDZ-binding motif of the MasR but by receptor coupling to Gαi-adenylyl cyclase signaling pathway. Incubation of MasR transfected cells with Ang-(1–7) or the synthetic ligand AVE 0991 amplified MasR negative modulation of cAMP levels. On the other hand, we provided evidence for lack of MasR-associated modulation of Ca^2+^ levels by Ang-(1–7). Finally, it was determined that the MasR attenuated Ang-(1–7)-induced ERK1/2 phosphorylation mediated by AT1R. We provided further characterization of MasR signaling mechanisms regarding its constitutive activity and response to putative ligands. This information could prove useful to better describe MasR physiological role and development of therapeutic agents that could modulate its action.

## Introduction

The Mas receptor (MasR) is a class A G protein-coupled receptor (GPCR) first described in 1986 because of its ability to transform and induce foci in NIH 3T3 cells and promote tumorigenicity in nude mice (Young et al., 1986). Currently, it is considered the founding member of the Mas-related GPCR family (Mrgpr), consisting of ∼40 orphan receptors structurally homologous to MasR expressed in specific subpopulations of sensory neurons that detect painful stimuli (Alenina et al., 2008). Although there has been a lot of information accumulated regarding its physiological function, based on the multiple criteria used to recommend the pairing of an orphan receptor with its cognate ligand, the IUPHAR/BPS Guide to Pharmacology still considers the MasR as an orphan GPCR (Davenport et al., 2013; Karnik et al., 2015).

Recently, we provided information that questions the determination of the endogenous levels of MasR protein through antibody-based techniques (Burghi et al., 2017). Thus, data from alternative methods such as mRNA determination appear so far, as the most solid in terms of MasR tissue distribution and abundance. In both rats and mice, the highest levels of MasR mRNA have been found in brain and testis (Young et al., 1988; Bunnemann et al., 1990; Martin et al., 1992; Metzger et al., 1995; Alenina et al., 2002). Lower levels of MasR transcripts have also been detected in heart and kidney (Metzger et al., 1995; Ferrario et al., 2005; Alenina et al., 2008; Burghi et al., 2017). Noteworthy, the phenotype of MasR knockout (MasR-KO) mice indicates that lack of MasR affects behavioral, cardiovascular, renal and metabolic processes. Both damaging and protective effects have been reported in MasR-KO mice (Karnik et al., 2015).

The MasR has been proposed as a candidate receptor for angiotensin (Ang)-(1-7) (Santos et al., 2003; Karnik et al., 2015), an endogenously produced heptapeptide that belongs to the renin-angiotensin system (RAS) (Ferrario and Iyer, 1998), mainly generated through the cleaving action of Ang-converting enzyme 2 (ACE2) on Ang II (Vickers et al., 2002; Oudit et al., 2003). Accumulating evidence indicates that the ACE2/Ang-(1–7) constitute a protective axis of RAS that counterbalances the pathophysiological effects of overactivation of the classical RAS axis formed by ACE, Ang II, and Ang II type 1 (AT1) receptor. Characterization of receptor components of the ACE2/Ang-(1–7 axis is still ongoing (Karnik et al., 2017). There are reports that have paired MasR with other peptides of the RAS, including Ang III, Ang IV (Gembardt et al., 2008) and angioprotectin (Jankowski et al., 2011), but also with the unrelated neuropeptide FF (Dong et al., 2001).

In many studies, the signaling pathways activated by MasR have been evaluated using the putative ligand Ang-(1–7). It has been shown that Ang-(1–7) stimulates arachidonic acid production in MasR transfected CHO and COS cells (Santos et al., 2003; Gembardt et al., 2008) and in human mesangial cells that express MasR (Zimpelmann and Burns, 2009). The actions of Ang-(1–7) have also been associated to the activation of mitogen-activated protein kinase (MAPK) p38 (Zimpelmann and Burns, 2009), modulation of the activity of ERK1/2 (Tallant and Clark, 2003; Gallagher and Tallant, 2004; Tallant et al., 2005; Su et al., 2006; Sampaio et al., 2007a; Zimpelmann and Burns, 2009; Liu et al., 2012), activation of phosphatidylinositol 3-kinase/Akt pathway (Giani et al., 2007; Sampaio et al., 2007b; Muñoz et al., 2010), and phospholipase A2 (Santos et al., 2003). Although the MasR belongs to the superfamily of GPCRs, Ang-(1–7) failed to produce a conventional G-protein signaling response as measured by the levels of second messengers like Ca^2+^, IP_3_, and cAMP in MasR transfected cells (Bikkavilli et al., 2006; Shemesh et al., 2008; Zhang et al., 2012; Tirupula et al., 2014). On the contrary, conventional G protein signaling has been reported upon stimulation with NPFF (Dong et al., 2001) and surrogate ligands (Bikkavilli et al., 2006; Shemesh et al., 2008; Savergnini et al., 2010; Tirupula et al., 2014). Synthetic non-peptide ligands of the MasR have been shown to modulate the Gα_q_-phospholipase C (PLC) pathway (Zhang et al., 2012).

As the MasR has been suggested to participate in several physiological processes, definitive elucidation of the signaling mechanisms involved in triggering its actions is essential to develop agents that could modulate its function. Accordingly, the aim of this work was to further characterize the signaling pathways triggered by the MasR. To do that, we measured cAMP and Ca^2+^ levels in both naïve and MasR transfected cells in basal conditions and upon incubation with putative MasR ligands. Besides, we evaluated activation of ERK1/2 by Ang-(1–7) in MasR transfected cells. Results indicated the existence of a high degree of MasR constitutive activity toward cAMP modulation. This effect was not mediated by the PDZ-binding motif of the MasR but by receptor coupling to Gαi-adenylyl cyclase signaling pathway. Modulation of cAMP levels was also observed after incubation with Ang-(1–7) and AVE 0991. We also provide evidence for lack of MasR-associated modulation of Ca^2+^ levels by Ang-(1–7). Finally, the MasR attenuated Ang-(1–7) induced ERK phosphorylation mediated by AT1R.

## Materials and Methods

### Chemicals

Angiotensin-(1-7), cell culture medium, bovine serum albumin (BSA), isobutyl methylxanthine (IBMX), cAMP, forskolin, Ang-1-7, FURA-2AM, histamine dihydrochloride, triton X-100, pertussis toxin and protease inhibitors were obtained from Sigma Chemical Company (St. Louis, MO, United States). Fetal calf serum was from Natocor (Argentina). [^3^H] cAMP, was purchased from PerkinElmer Life Sciences (Boston, MA, United States). AVE 0991: 5-formyl-4-methoxy-2-phenyl-1-[[4-[2-ethylaminocarbonylsulfonamido-5-isobutil-3-thienyl] phenyl]-methyl]imidazole was obtained from ApexBio Technology LLC (Houston, TX, United States). Other chemicals used were of analytical grade and obtained from standard sources.

### Plasmid Constructions

A pcDNA3.1(+)-myc-MasR expression construct corresponding to the human MasR (accession number: NM_002377.2) fused to the c-myc tag peptide at N-terminal was acquired from Life Technologies (Grand Island, NY, United States). An empty vector [pcDNA 3.1 (+)] was used both as negative control and to deliver an equal amount of plasmid per transfection in all the experiments. A pcDNA3.1(+)-myc-MasR-ΔPDZ expression construct corresponding to the human MasR, fused to the c-myc tag peptide at N-terminal, and lacking the last five amino acids (Val-Glu-Thr-Val-Val) was acquired from Life Technologies (Grand Island, NY, United States). A pZN-Gα_i2_ plasmid coding for rat Gα_i2_ was a generous gift from Dr. J. S. Gutkind (Moores Cancer Center, University of California, San Diego).

### Cell Culture and Transfections

HEK293T (human embryonic kidney) and A549 (human lung carcinoma) cells were cultured in Dulbecco’s modified Eagle’s medium (DMEM) supplemented with 10% fetal calf serum and 50 μg/ml gentamicin. Cultures were maintained at 37°C in humidified atmosphere containing 5% CO_2_. For transient transfections, cells were grown to 80–90% confluency and the cDNA constructs were transfected using the K2 Transfection System (Biontex, Munich, Germany). The transfection protocol was optimized as recommended by the suppliers. Assays were performed 48 h after transfection. For RNA interference experiments, cells were grown to 80–90% confluency and were cotransfected with pcDNA 3.1 or pcDNA 3.1/myc-MasR plasmids and Non-Targeting siRNA (UAAGGCUAUGAAGAGAUAC) or human AT1R siRNA (AUACGUGACUGUAGAAUUG) (100 nM) (Horizon Discovery Ltd., Cambridge, United Kingdom) using the K2 Transfection System. The transfection protocol was optimized as recommended by the suppliers. Assays were performed 48 h after transfection.

### Western Blot Assay

In Western blot assays directed to determine ERK1/2 phosphorylation levels, cells were serum starved for 8 h. In concentration-response assays, cells were incubated with the indicated concentrations of Ang (1–7) for 5 min; in time-course experiments, cells were incubated at the indicated periods of time with Ang (1–7) at a final concentration of either 1.10^-10^, 1.10^-9^, or 1.10^-7^ M. After stimulation cells were washed and lysed in lysis buffer [1% Triton, 100 mM Hepes, 100 mM sodium pyrophosphate, 100 mM sodium fluoride, 10 mM EDTA, 10 mM sodium vanadate, 2 mM phenylmethylsulfonyl fluoride (PMSF) and 0.035 trypsin inhibitory units/ml aprotinin (pH 7.4)] and sonicated to shear DNA. Resulting homogenates were centrifuged at 15,700 g for 30 min at 15,700 g to remove insoluble material. Protein concentration in the supernatant was determined by the BCA assay (Pierce BCA Protein Assay Kit, Thermo Fisher Scientific, MA, United States). Equal amounts of proteins were denatured by boiling 5 min in Laemmli buffer (50 mM Tris–HCl pH 6.8, 2% SDS, 100 mM 2-mercaptoethanol, 10% glycerol and 0.05% bromophenol blue). In Western blot assays directed to determine MasR protein expression levels, cells were washed, lysed at room temperature in Laemmli buffer and sonicated to shear DNA. Heat treatment of samples was avoided to prevent aggregation of overexpressed MasR. Total cell lysates were resolved by SDS-PAGE, blotted and incubated with primary antibodies anti-myc (cat# 2272), anti-pERK1/2 (Thr202/Tyr204) (cat# 9101), anti-ERK1/2 (cat# 9102) (Cell Signaling Technology, Beverly, MA, United States) or anti-βtubulin (cat# 6046) (Abcam, Cambridge, MA, United States). After incubation for 1 h at room temperature with a horseradish peroxidase-conjugated anti-rabbit antibody (cat# sc-2004) (Santa Cruz Biotechnology, Santa Cruz, CA, United States), reaction products were revealed by enhanced chemiluminescence (ECL Plus, Pierce, Rockford, IL, United States).

### cAMP Assay

For determination of cAMP basal levels, transfected cells were seeded in 12 wells dishes, 24 h later 100% confluent cells were starved for 2 h and pretreated, when indicated, for 1 h with pertussis toxin (100 ng/ml) and incubated later for 30 min in basal culture medium supplemented with 1 mM IBMX at 37°C. For time-course assays, transfected cells were seeded in 24 wells dishes and after 24 h, 100% confluent cells were starved for 2 h and incubated 5 min in basal culture medium supplemented with 1 mM IBMX at 37°C, followed by exposure to 1 μM forskolin for 5, 10, 15, 30, or 60 min. In concentration response assays, transfected cells were seeded in 24 wells dishes after 24 h, 100% confluent cells were starved for 2 h and incubated 5 min in basal culture medium supplemented with 1 mM IBMX at 37°C, followed by 10 min exposure to 1 μM forskolin to increase basal cAMP levels, and incubated for 5 min with indicated concentrations of Ang-(1–7) or AVE 0991. For all cAMP determinations, the reactions were stopped by ethanol addition followed by centrifugation at 2,000 *g* for 10 min. The ethanol phase was then dried, and the residue resuspended in 50 mM Tris-HCl pH 7.4, 0.1% BSA. cAMP content was determined by competition of [^3^H]cAMP for PKA, as previously described (Davio et al., 1995).

### Cell Proliferation Assay

Cell proliferation was determined by a colorimetric assay using Cell Titer 96 AQueous Non-Radioactive Cell Proliferation Assay (Promega, Madison, WI, United States) according to the manufacturer’s instructions. Transfected cells were seeded at 2.0 × 10^4^ cells/well in a 96-wells plate and incubated in an atmosphere of 5% CO_2_ at 37°C. After incubation for 48 or 72 h, 20 μl of MTS was added to each well and further incubated for 2 h at 37°C. The absorbance was measured at 490 nm using the FlexStation 3 microplate reader (Molecular Devices Inc., San Jose, CA, United States).

### Ca^2+^ Measurements

Changes in intracellular Ca^2+^ concentration were measured using fura-2 acetoxymethyl ester (Fura-2AM) fluorescent indicator. A549 cells were seeded in 96 wells dishes for 24 h (90–100% confluence). Thereafter, culture media was replaced by loading buffer (140 mM NaCl, 3.9 mM KCl, 0.7 mM KH_2_PO_4_, 0.5 mM Na_2_HPO_4_, 1 mM CaCl_2_, 0.5 mM MgCl_2_ 10 mM glucose, 0.1% BSA, 20 mM HEPES, pH 7.4) containing 4 μM Fura-2AM and 0.2% pluronic acid and cells were incubated for 90 min at 37°C in humidified atmosphere containing 5% CO_2_ to facilitate the hydrolysis of the ester to the acid form. Excess dye was removed by washing cells with loading buffer. Fluorescence was measured in a FlexStation 3 microplate reader (Molecular Devices Inc., San Jose, CA, United States). The wavelength was set at 340 and 380 nm, and detection was at 500 nm. After 30 s of initial recording to determine basal levels, 100 nM Ang (1–7) or 100 μM histamine was added in 100 μl final volumes, and the time course of intracellular Ca^2+^ mobilization was recorded for 180 s. At the end of the time course, TritonX-100 (0.25% v/v) was added to determine *F*_max_. Autofluorescence was quantified by measuring the fluorescence produced by an equivalent suspension of not-loaded cells. Results were expressed as the ratio of fluorescence’s 340/380 over basal. Basal levels were determined as the media of the recorded measurements in the first 30 s for each well.

### RT-PCR and Quantitative RT-PCR

Total RNA was isolated from wild type or transfected HEK293T cells using Quick-Zol reagent (Kalium Technologies, Bernal, Argentina) following the manufacturer’s instructions. For the first-strand cDNA synthesis, 1 μg of total RNA was reverse-transcribed using the High Capacity cDNA Reverse Transcription kit (Applied Biosystems^TM^, Beverly, MA, United States) with random primers. Quantitative real-time PCR (qPCR) was performed in triplicate on the Rotor Gene Q cycler (Qiagen, Hilden, Germany) using the resulting cDNA, the HOT FIREPol EvaGreen qPCR Mix Plus (Solis BioDyne, Tartu, Estonia) for product detection, and the following primers: human MasR (NM_002377.2) forward, 5′-ATTCCTCATCTTCGCTATGCC -3′ and reverse 5′-GCAGGGAAATGTGGTGTAGG-3′; human AT1R (M93394.1) forward, 5′-CCCAAAATTCAACCCTTCCG-3′ and reverse, 5′-CAGAAAAGGAAACAGGAAACCC-3′; human AT2R (NM_000686.4) forward, 5′-CCCTGAACATGTTTGCAAGC-3′ and reverse 5′-AGGGGTAGATGACAGATTGG-3′; and human β-Actin forward, 5′-GGACTTCGAGCAAGAGATGG-3′ and reverse 5′-AGCACTGTGTTGGCGTACAG-3′. The cDNA was amplified by 45 cycles of denaturing (15 s at 95°C), annealing (30 s at 60°C), and extension (30 s at 72°C) steps. The specificity of each primer set was monitored by analyzing the dissociation curve, and the relative MasR, AT1R or AT2R mRNA quantification was performed using the comparative ΔΔCt method using β-Actin as the housekeeping gene.

### Statistical Analysis

Experiments were run as many times as indicated in legends for figures and data are presented as mean ± standard error of the mean (SEM). Statistical analysis were carried out by two-sided Students’ *t*-test, or one-way- or two-way-ANOVA, followed by Tukeys’ post test. *Post hoc* tests were run only if overall statistically significant difference between means were obtained. Statistical significance was accepted when *P* < 0.05. No statistical differences between variances were observed along the whole work according to Brown–Forsythe test. All analysis were carried out with GraphPad Prism 6.00 for Windows, GraphPad Software.

## Results

### Modulation of Basal cAMP Levels by the MasR

**Figure 1:**
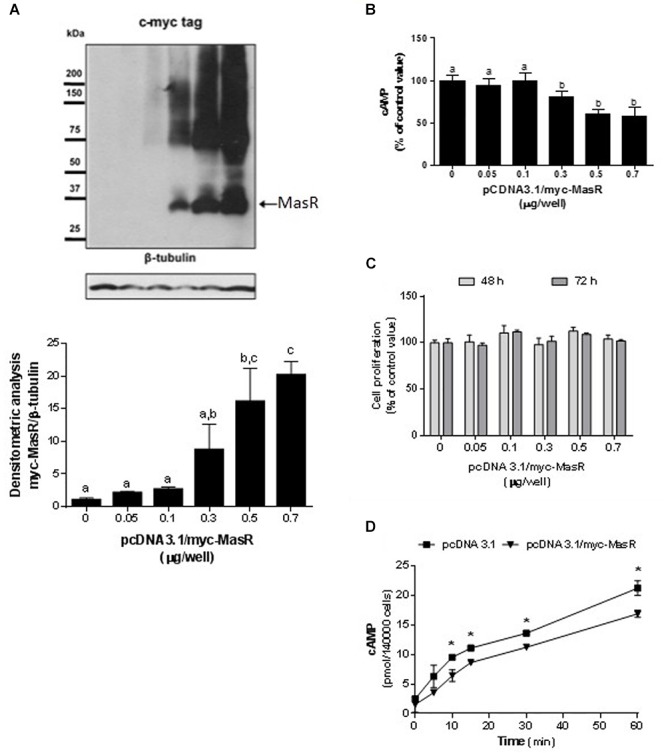
Constitutive activity of MasR in HEK293T transfected cells. **(A)** Analysis of MasR expression by Western Blot. Cells were transfected with the indicated amounts of a plasmid encoding the c-myc tagged MasR (pcDNA3.1/c-myc-MasR) and cell extracts were resolved by SDS-PAGE and Western Blot using anti-c-myc antibody. Detection of β-tubulin was used as a protein loading control. Blots were subjected to densitometry analysis using ImageJ software. Data are presented as mean ± SEM respect to control of at least three independent experiments. Images are representative of at least three independent experiments. Different letters denote significant difference at *p* < 0.05. **(B)** cAMP accumulation. Cells were transfected with the indicated amounts of pcDNA3.1/c-myc-MasR and cAMP levels were determined after incubation for 30 min with IBMX. 100% corresponds to cAMP levels in cells transfected with mock (pcDNA3.1). Data represent mean ± SEM of four independent experiments. Different letters denote significant difference at *p* < 0.05. **(C)** Cell proliferation. Cells transfected with the indicated amounts of pcDNA3.1/c-myc-MasR were cultured for 48 h or 72 h. Cell viability was determined by MTS assay and reported as a percent of proliferation respect to mock transfected cells (*n* = 3). **(D)** Time-course production of cAMP. Cells transfected with 0.5 μg/well pcDNA3.1/c-myc-MasR were stimulated during the indicated times with 1 μM forskolin in the presence of IBMX 1 mM and cAMP levels were determined. Data represent mean ± SEM of three independent experiments. Asterisk denote significant difference at *p* < 0.05 by plasmid type within the same time point.

We initiated the study of the signaling pathways associated with the MasR by evaluating whether MasR itself modulates the intracellular levels of cAMP either through the activation or inhibition of the adenylate cyclase. HEK293T cells were transfected with different amounts of pcDNA 3.1/myc-MasR plasmid. Protein expression of MasR increased in response to increasing amounts of the pcDNA 3.1/myc-MasR plasmid used for transfections (Figure 1A) and showed an inverse relationship with accumulated cAMP levels (Figure 1B). Cell viability was not affected after transfecting cells with the pcDNA 3.1/myc-MasR plasmid (Figure 1C). Additionally, cAMP was measured after incubating cells with the adenylate cyclase activator, forskolin, for various periods of time. Under this experimental condition, cells that overexpressed the MasR accumulated significantly lower levels of cAMP compared to control cells (Figure 1D).

**Figure 2:**
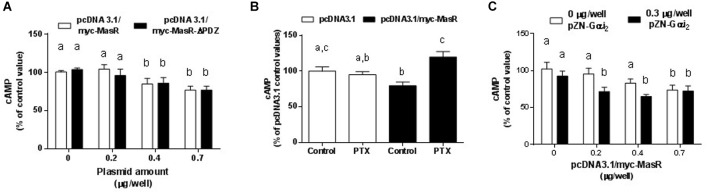
Molecular mechanisms of MasR constitutive activity. **(A)** Effect of deletion of the PDZ- binding motif. Cells were transfected with pcDNA3.1/c-myc-MasR or a plasmid lacking the last 5 amino acids including the PDZ-binding motif. Basal cAMP levels were determined after 30 min incubation with IBMX. 100% corresponds to cAMP levels in cells transfected with the empty vector. Data represent mean ± SEM of three independent experiments. Different letters denote significant difference at *p* < 0.05. **(B)** Effect of treatment with pertussis toxin. Basal cAMP levels were determined after 30 min incubation with IBMX in cells treated with PTX 100 ng/mL or vehicle for 1 h. 100% corresponds to cAMP levels in cells transfected with the empty vector and incubated with vehicle. Data represent mean ± SEM of four independent experiments. Different letters denote significant difference at *p* < 0.05. **(C)** Effect of Gαi overexpression. Cells were transfected with the indicated amounts of pcDNA3.1/c-myc-MasR and empty vector or 0.3 μg/well of pZN-Gαi_2_. Basal cAMP levels were determined after 30 min incubation with IBMX. 100% corresponds to cAMP levels in cells transfected with the empty vector. Data represent mean ± SEM of three independent experiments. Different letters denote significant difference at *P* < 0.05.

The potential participation of the PDZ-binding motif of the MasR (ETVV) in the regulation of basal cAMP levels was evaluated by transfecting HEK293T cells with pcDNA 3.1/myc-MasR or pcDNA3.1/myc-MasR-ΔPDZ constructs. As shown in Figure 2A, the ability of the MasR to decrease cAMP levels was not affected by elimination of the PDZ-binding motif, since the decrease in cAMP levels detected in cells that expressed the MasR lacking the PDZ-binding motif was similar to that detected in cells expressing the wild type MasR.

To investigate the signaling pathways by which the MasR induced a reduction in cAMP cellular levels, we evaluated the effect of MasR expression on the accumulation of cAMP either in the presence of pertussis toxin (PTX) or after co-expressing the MasR with Gαi_2_ protein. Pretreatment with PTX reversed the decrease in cAMP levels associated with MasR overexpression (Figure 2B) whereas no effect was observed in cells transfected with the empty vector. As shown in Figure 2C, overexpression of Gαi_2_ alone in HEK293T cells caused a slight decrease in cAMP levels possibly due to its association with endogenous Gαi-coupled GPCRs. When pcDNA 3.1/myc-MasR was co-transfected with the pZN-Gαi_2_ plasmid, cAMP levels were significantly lower than those obtained after transfecting with the pcDNA 3.1/myc-MasR plasmid alone and similar to those obtained with the highest amount of pcDNA 3.1/myc-MasR employed (0.7 μg/well). No significant differences in cAMP levels were found between transfecting 0.7 μg/well of pcDNA 3.1/myc-MasR alone or with pZN-Gαi_2_ plasmid. Thus, this condition could indicate the maximal detectable effect of MasR overexpression over cAMP basal levels (Figure 2C).

### MasR Responses to Ang-(1–7) and AVE 0991

**Figure 3:**
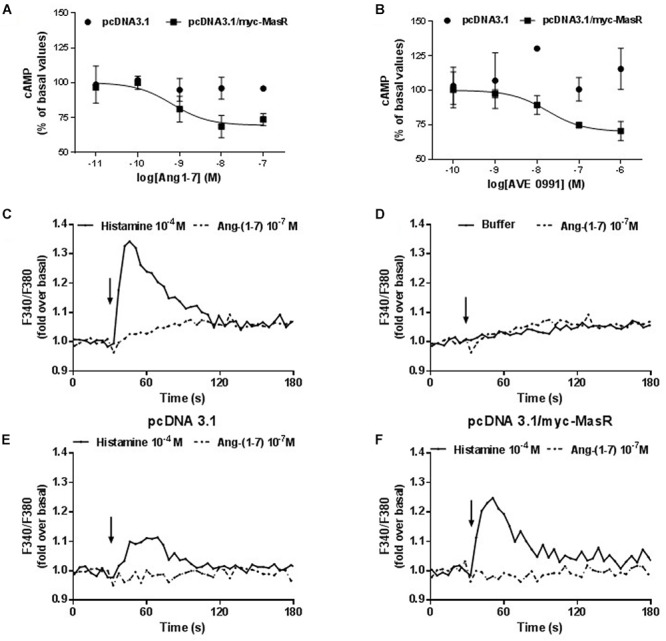
Effect of Ang-1–7 and AVE 0991 on cAMP and Ca^2+^ levels in MasR-expressing cells. Cells transfected with pcDNA3.1/c-myc-MasR or pcDNA3.1 were incubated with increasing concentrations of Ang-(1–7) **(A)** or AVE 0991 **(B)** in presence of 1 mM IBMX and 1 μM FSK. The levels of cAMP were determined after 5 min 100% corresponds to basal levels of cAMP. Data represent mean ± SEM of three independent experiments. Variation of the intracellular Ca^2+^ concentration in A549 cells in response to Ang-(1–7). A549 cells with endogenous expression of MasR **(C,D)** or transfected either with the empty pcDNA 3.1 vector **(E)** or with the pcDNA 3.1/myc-MasR vector **(F)** were placed in 96-well plates 24 h before the experiment. Cells were incubated with FURA 2-AM, washed and stimulated with Histamine 10^-4^ M (positive control) and Ang-(1–7) 10^-7^ M **(C–F)**. Arrows indicate addition of the stimulus. Fluorescence determinations were performed as described in Section “Materials and Methods.” Results are representative of three independent experiments.

The effect of putative MasR ligands on the modulation of cAMP levels was evaluated using HEK293T cells overexpressing MasR that were incubated with different concentrations of either Ang-(1–7) or the synthetic non-peptide ligand AVE 0991 (Figure 3A,B). Incubation with Ang-(1–7) or AVE 0991 led to a concentration-dependent decrease in the production of cAMP (pEC50 = 9.18 ± 0.32 and 7.75 ± 0.40, respectively). In cells transfected with the empty vector, incubation with either ligand did not alter cAMP levels (Figure 3A,B).

In addition, using a lung carcinoma cell line (A549) that expresses the MasR endogenously (Gallagher and Tallant, 2004), we investigated whether Ang-(1–7) can modulate the intracellular Ca^2+^ levels (Figure 3C–F). Incubation with Ang-(1–7) at 10^-7^ M concentration did not modify intracellular Ca^2+^ levels in A549 cells (Figure 3D). Unlike Ang-(1–7), histamine (10^-4^ M) (used as positive control) (Zappia et al., 2015), originated a significant increase in cytosolic Ca^2+^ levels (Figure 3C). To evaluate whether the lack of response to Ang-(1–7) was a consequence of a low level of endogenous MasR expression, the same experiments were performed on A549 cells transfected with the pcDNA 3.1/myc-MasR plasmid. Despite overexpression of the MasR, stimulation with 10^-7^ M Ang- (1–7) did not modify intracellular Ca^2+^ levels in A549 cells. Under the same conditions histamine led to an important increase in Ca^2+^ levels (Figure 3E,F).

**Figure 4:**
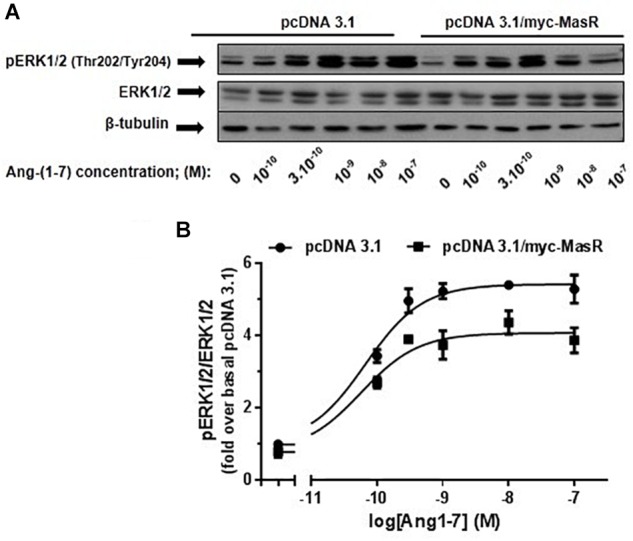
Dose-response effect of Ang-(1–7) on ERK1/2 phosphorylation levels. HEK293T cells were transfected either with the pcDNA3.1/myc-MasR vector or with the empty vector (pcDNA 3.1), starved in DMEM without serum for 8 h and then incubated for 5 min with increasing concentrations of Ang-(1–7). **(A)** Phosphorylation levels of ERK1/2 at activating residues Thr202/Tyr204, total ERK1/2 and β-tubulin abundance were determined by Western Blotting. **(B)** Data quantification. Bands intensity was quantified by optical densitometry, each individual value of pERK was normalized to that of total ERK and expressed as relative to the value obtained after incubation with cells transfected with pcDNA 3.1 and incubated with vehicle (basal value). Data are means ± SEM of three independent experiments. Values were fitted to a sigmoidal dose-response curve. Maximal response best-fitted values were analyzed by extra sum-of-squares *F* test, significantly different (*P* < 0.001).

Taking into account the relevance of the MAPKs ERK1/2 in the signaling pathways modulated by GPCRs and considering that, on this pathway, Ang-(1–7) has been shown to display opposite actions (Tallant and Clark, 2003; Gallagher and Tallant, 2004; Tallant et al., 2005; Su et al., 2006; Sampaio et al., 2007a; Zimpelmann and Burns, 2009; Liu et al., 2012), it was of great interest to study the modulation of ERK1/2 signaling pathway by this peptide. Thus, in HEK293T cells, we determined the phosphorylation levels of ERK1/2 in response to incubation with different concentrations of Ang-(1–7) (Figure 4). In cells transfected with the pcDNA 3.1/myc-MasR plasmid incubation with Ang-(1–7) led to a concentration-dependent increase in ERK1/2 phosphorylation levels. Unexpectedly a similar result was found in cells that had been transfected with the empty vector. Suggestively, the presence of the MasR resulted in lower ERK1/2 activation at every concentration of Ang-(1–7) analyzed (Figure 4).

**Figure 5:**
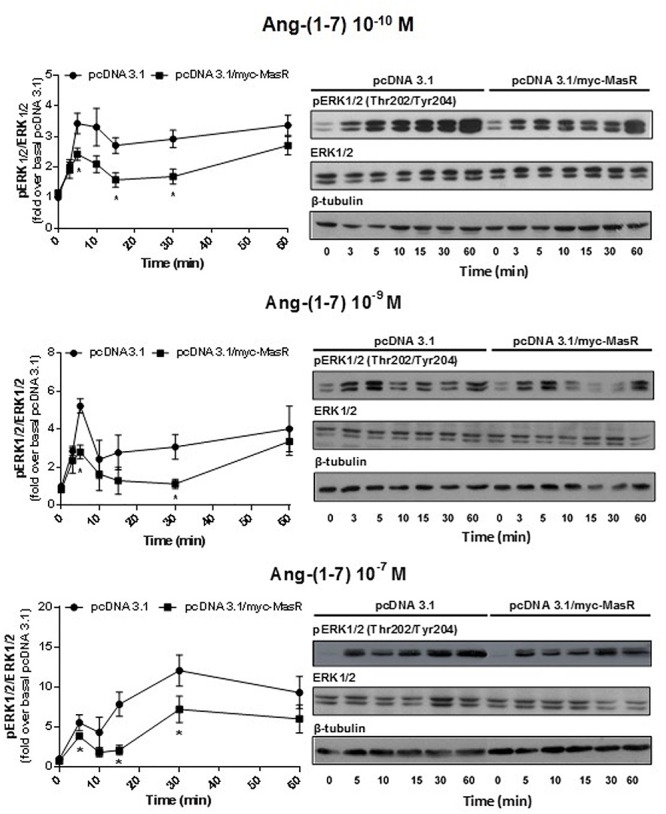
Kinetics of Ang-(1–7)-induced ERK1/2 phosphorylation. HEK293T cells transfected as described in Figure 4, were incubated with Ang-(1–7) at the different concentrations indicated for the indicated periods. Phosphorylation levels of ERK1/2, total ERK1/2, and β-tubulin were determined by Western Blotting. Phosphorylation of ERK1/2 was normalized to that of total ERK1/2 and expressed as relative to the value obtained at 0 min in cells transfected with the empty vector (pcDNA 3.1). Data represents the mean ± SEM of three independent experiments. ^∗^*P* < 0.05 by plasmid type within the same time point.

Next, we performed a time-response analysis of ERK1/2 phosphorylation levels using three concentrations of Ang-(1–7): 10^-10^, 10^-9^, and 10^-7^ M (Figure 5). Incubation with Ang-(1–7) at 10^-10^ and 10^-9^ M concentration induced similar patterns of ERK1/2 phosphorylation. At 10^-10^ and 10^-9^ M Ang-(1–7), ERK1/2 phosphorylation peaked at 5 min, decreased and peaked again at 60 min (Figure 5). In contrast, incubation with a 10^-7^ M Ang-(1–7) resulted in a peak of ERK1/2 phosphorylation at 30 min (Figure 5).

At every concentration of Ang-(1–7) analyzed, the presence of the MasR did not modify the patterns of ERK phosphorylation obtained. However, in concordance with results shown in Figure 4, it was evident that in MasR-transfected cells, lower ERK1/2 phosphorylation levels were attained compared to those obtained in cells transfected with the empty vector regardless the concentration of Ang-(1–7) that was present during the assay (Figure 5).

### mRNA Levels of AT1R, AT2R, and MasR in HEK293T Cells

**Figure 6:**
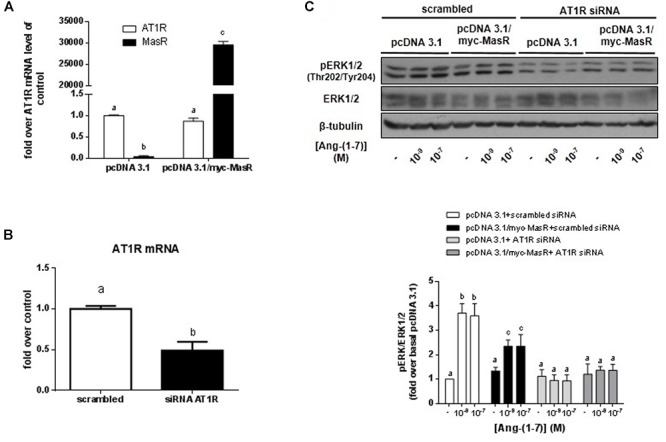
Participation of the MasR and AT1R in Ang-(1–7) induced ERK phosphorylation. **(A)** Levels of mRNA coding for AT1R and MasR in HEK293T cells. The levels of mRNA were determined by qPCR in HEK293T cells transfected with pcDNA 3.1 and pcDNA 3.1/myc-MasR vectors as described in Section “Materials and Methods.” Data represent the mean ± SEM of three independent experiments. **(B)** Levels of mRNA coding for AT1R in cells transfected with AT1R siRNA. HEK293T cells were transfected with scrambled siRNA or AT1R siRNA and after 48 h the mRNA levels coding for AT1R were determined by qPCR as described in Section “Materials and Methods.” Data represent the mean ± SEM of three independent experiments. **(C)** Effect of silencing the AT1R on Ang-(1–7)-induced activation of ERK1/2. HEK293T cells that were transfected with the pcDNA3.1/myc-MasR plasmid or with the empty vector (pcDNA 3.1), were incubated for 5 min with Ang-(1–7) at the indicated concentrations. Experiments were performed in AT1R-silenced cells and in control cells (scrambled siRNA). Phosphorylation levels of ERK1/2 at activating Thr202/Tyr204, total ERK1/2 and β-tubulin abundance were determined by Western Blotting. Bands intensities were quantified by optical densitometry, pERK individual values were normalized to total ERK1/2 values and expressed as relative to the value obtained with control cells (transfected with pcDNA 3.1 and scramble siRNA) and incubated with vehicle (basal value). Data are means ± SEM of three independent experiments. Different letters denote significant difference at *P* < 0.05.

The levels of mRNA coding for endogenous receptors that could potentially interact with Ang-(1–7) were determined in HEK293T cells by qPCR. As shown in Figure 6, HEK293T cells endogenously express the AT1R. In comparison with AT1R levels, MasR levels were barely detectable, while the presence of AT2R mRNA could not be detected. After transfecting HEK293T cells with pcDNA 3.1/myc-MasR, mRNA levels coding for the MasR were approximately 30,000 times higher than that encoding for the AT1R. There was no apparent modification in AT1R mRNA levels after transfection with the myc-MasR plasmid (Figure 6A).

### Participation of MasR and AT1R in Ang-(1–7) Induced ERK1/2 Phosphorylation

To evaluate the possible participation of the AT1R in Ang-(1–7)-induced ERK1/2 activation, we silenced the expression of this receptor in HEK293T cells using an AT1R siRNA (Figure 6B). After 48 h of transfection, the mRNA levels of AT1R were ∼50% lower in AT1RsiRNA-transfected cells as compared to those transfected with scrambled siRNA (Figure 6B).

In concordance with results shown in Figure 4, in cells cotransfected with the empty vector and scrambled siRNA, incubation with 10^-9^ and 10^-7^ M Ang-(1–7) for 5 min led to a significant increase in ERK1/2 phosphorylation levels compared to the basal value. Cotransfection of pcDNA 3.1/myc-MasR with scrambled siRNA resulted in lower ERK1/2 activation at both concentrations of Ang-(1–7) analyzed (Figure 6C). However, after silencing the AT1R, no response to Ang-(1–7) was observed. Ang-(1–7)-induced ERK1/2 phosphorylation levels attained under these conditions were similar to those obtained after incubation with vehicle regardless the plasmid that was used for transfection (Figure 6C).

## Discussion

To date, more than 100 GPCRs have no known endogenous or natural exogenous ligands, suggesting that many biological functions of these receptors are still unknown. These orphan GPCRs have immense therapeutic potential, since ∼30% of all clinically relevant drugs modulate the activity of GPCRs or related signaling pathways (Solinski et al., 2014; Fang et al., 2015; Hauser et al., 2018). Particularly for MasR, numerous studies have attributed a role for this receptor in the regulation of cardiovascular, renal and central nervous system (CNS) function (Karnik et al., 2015). However, the intracellular signaling pathways activated by MasR remain insufficiently characterized, preventing definitive knowledge of ligand/receptor pharmacology.

In the current study, we analyzed whether the MasR is capable of modulating the intracellular levels of cAMP in the absence of an external stimulus. In order to determine the constitutive activity of the MasR we increased the number of receptors expressed by varying the amount of the pcDNA 3.1/myc-MasR plasmid used in the transfections. The decrease in cAMP basal levels associated with the increased MasR expression indicated that MasR possesses constitutive activity toward negative modulation of cAMP levels. Moreover, this was also detected by the addition of the activator of adenylate cyclase forskolin. The decrease in cAMP accumulation in basal conditions and/or stimulated by forskolin has been reported in studies of constitutive activity of GPCRs that are coupled to Gαi/o proteins (Seifert and Wenzel-Seifert, 2002) or that form complexes with proteins with PDZ domains (Broselid et al., 2014). Recently, the interaction of the c-terminal binding motif of the MasR with PDZ domains of different proteins has been reported (Tirupula et al., 2015). Considering this, we explored whether signaling pathways involving Gαi/o proteins and/or PDZ domains are associated with MasR and could lead to a decrease in cAMP levels. Lack of involvement of the PDZ-binding motif in the constitutive activity of the MasR in terms of inhibition of cAMP levels is in agreement with a recent work in which deletion of this motif did not modify the constitutive activity of MasR in terms of stimulation of inositol-1-phosphate pathway (Tirupula et al., 2015). Possibly proteins with PDZ domains are involved only in regulating the ubiquitination and degradation of the MasR (Bian et al., 2013).

Given that PTX not only prevents the stimulation of Gαi/o proteins by GPCRs bound to agonists, but also that exerted by those free of ligands, it is a very useful tool to highlight the constitutive activity of many GPCRs (Seifert and Wenzel-Seifert, 2002). Thus, the reversal of the decrease in cAMP levels obtained by preincubating the cells overexpressing MasR with PTX suggested that this effect is mediated by Gαi/o proteins. In addition, it was shown that the concomitant overexpression of the Gαi_2_ protein caused a greater decrease in basal cAMP levels. By modifying the stoichiometry of other of the components of the system, in this case, by increasing the Gαi_2_ protein expression levels, we facilitated receptors to reach active conformations coupled to the G protein by which they signal. This gives further support to the MasR having constitutive activity. In all, these results indicated that in the absence of an external stimulus, MasR is capable of coupling and activating the Gαi protein with the consequent decrease in cAMP levels. Previous studies reported that MasR constitutively activates Gαq and Gα_11_ proteins (Canals et al., 2006; Zhang et al., 2012; Tirupula et al., 2014). In the same system, Tirupula et al. (2014) also reported constitutive coupling of MasR to Gα_12_ and even Gαs proteins. Interestingly, compound AR234960, reported as an agonist for the Gαq-mediated pathway, was shown to decrease cAMP levels (Zhang et al., 2012; Tirupula et al., 2014).

The physiological relevance of the constitutive activity of the MasR remains to be determined. Constitutive activity of GPCRs for various neurotransmitters contributes to basal neuronal activity (Seifert and Wenzel-Seifert, 2002). The mRNA encoding the MasR has been detected in various regions of the rat and mouse brain (Young et al., 1988; Bunnemann et al., 1990; Martin et al., 1992; Metzger et al., 1995). In addition, it has been reported that MasR-KO mice have alterations of the CNS (Walther et al., 1998, 2000a,b). Thus, the constitutive activity of the MasR could play a regulatory role in this system. In addition, when analyzing the control of cardiac function by the autonomous system, it has been hypothesized that the Gαi protein could have a cardioprotective role (Foerster et al., 2003). MasR mRNA has been detected in rat and mouse heart (Metzger et al., 1995; Ferrario et al., 2005; Alenina et al., 2008; Burghi et al., 2017) and the phenotype of MasR-KO mice includes cardiac dysfunction (Santos et al., 2006; Gava et al., 2012). Thus, the constitutive activation of the Gαi protein mediated by the MasR may have a role in MasR-mediated cardioprotection. However, when interpreting the *in vivo* data, the contribution of Ang-(1–7) and other ligands cannot be ruled out. Indeed, the pathophysiological relevance of Ang-(1–7) in the heart has been highlighted by studies demonstrating that Ang-(1–7) can reduce or prevent cardiac remodeling by decreasing hypertrophy and fibrosis (Benter et al., 2006; Grobe et al., 2007). Additionally, several studies demonstrated that Ang-(1–7) exerts a protective effect against cardiac ischemia-induced injury and arrhythmias (Ferreira et al., 2001; De Mello, 2004). Moreover, given the beneficial effects obtained in heart dysfunction with either the orally active non-peptide compound AVE 0991 (Ferreira et al., 2007) or the surrogate ligand CGEN-856S (Savergnini et al., 2013), potential therapeutic applications of the findings obtained with Ang-(1–7) have been reinforced.

It is important to highlight the relevance of detecting the constitutive activity of orphan GPCRs, e.g., the MasR, as it allows posterior evaluation of the response of various ligands, putative or new, endogenous or synthetic, and with different efficacy, i.e., not only agonists and antagonists but also inverse agonists. This greatly contributes to the study of the MasR function(s) even in the absence of definitive knowledge of its endogenous ligand.

The decrease in cAMP levels in a concentration-dependent manner observed after incubation with Ang-(1–7) or AVE 0991 in MasR-overexpressing cells indicates that these ligands can act as MasR agonists for this signaling response. The absence of modulation of cAMP in the control cells agrees with the lack of significant expression levels of endogenous MasR and indicated that none of these ligands was able to activate this signaling pathway by binding to other endogenous receptors present in HEK293T cells.

Angiotensin-(1–7) has been reported to interact with the AT1R (Gironacci et al., 1999; Galandrin et al., 2016; Teixeira et al., 2017). Some of its actions appear to require the participation of the AT1R (Jaiswal et al., 1991; Osei et al., 1993; Garcia and Garvin, 1994; Galandrin et al., 2016; Teixeira et al., 2017), the AT2R (De Souza et al., 2004; Walters et al., 2005; Shimada et al., 2015) or both (Jaiswal et al., 1992; Gironacci et al., 1994). Since HEK293T cells endogenously express AT1R it was hypothesized that Ang-(1–7) could potentially modulate G proteins associated with AT1Rs. However, this was not the case. Our observations are in good correlation with previous reports showing that stimulation of HEK293T cells with Ang-(1–7) does not activate G proteins through endogenously expressed receptors (Galandrin et al., 2016; Teixeira et al., 2017). Although it was reported that Ang-(1–7) could bind to AT1Rs and promote the recruitment of β2-arrestin in HEK293T cells overexpressing AT1Rs, this interaction did not show efficacy toward modulation of G protein-mediated pathways (Galandrin et al., 2016; Teixeira et al., 2017).

Recently, Gaidarov et al. (2018) reported no change in forskolin-stimulated cAMP levels after incubation with Ang-(1–7) or AVE 0991 in MasR-transfected HEK293 cells whereas the small synthetic molecule AR234958 was shown to elicit a dose-dependent reduction of forskolin-stimulated cAMP in the same cells. On the contrary, we detected a decrease in cAMP levels in a concentration-dependent manner after incubation with Ang-(1–7) and AVE 0991. The reasons for this discrepancy could rely on the different incubation times used to detect the cAMP levels. As compared to the study of Gaidarov et al. (2018), pre-incubation of cells with forskolin was done for 10 min (instead of 30 min) and incubation with ligands in the presence of forskolin was done for 5 min (instead of 60 min). We designed and performed our experiments to detect the modulation of cAMP levels elicited by Ang-(1–7) and AVE 0991 after incubation for short periods of time to prevent as much as possible the proteolysis of Ang-(1–7). This experimental design also aimed at avoiding the mechanisms that tend to return the intracellular cAMP levels to basal values and the attenuation mechanisms of GPCRs signaling that could occur when incubating for longer periods of time. Finally, the cells were exposed to a moderate concentration of forskolin (1 μM) for a total time of 15 min (instead of 90 min) in order to avoid overstimulation of adenylyl cyclase reaching extremely high cAMP levels that could mask the reduction of this second messenger levels elicited by these ligands. This is of relevance considering that Ang-(1–7) and AVE 0991 may display lower efficacy than the synthetic ligand AR234958 toward this signaling pathway.

In contrast to our current results, incubation with Ang-(1–7) has been reported to increase cAMP levels in human umbilical vein endothelial cells, kidney mesangial cells, aortic vascular smooth muscle cells and in HEK293 cells overexpressing Mas or MrgD receptors (Tetzner et al., 2016). The latter response was blocked by D-Ala7-Ang-(1–7) and D-Pro7-Ang-(1–7); was also reduced in MasR- or MrgD- deficient cells and blunted in MasR/MrgD double knockout cells (Tetzner et al., 2016). The reasons for this discrepancy could rely on the different approaches used to detect the cAMP levels. We designed and performed our experiments in order to determine total cAMP cellular levels and not only the intracellular ones. This is of relevance since the intracellular regulation of cAMP depends not only on the balance between its production by adenylyl cyclase and its degradation by phosphodiesterases, but also on the efflux of cAMP through members of the multidrug associated resistance protein family (Carozzo et al., 2015).

Under the analyzed conditions, it was evident that Ang-(1–7) was not capable of activating the Gαq-PLC signaling pathway that leads to an increase in intracellular Ca^2+^ levels. Current results agree with previous reports that showed that unlike the effect exerted by synthetic MasR agonists, Ang-(1–7) appears not to modulate this pathway (Bikkavilli et al., 2006; Shemesh et al., 2008; Zhang et al., 2012; Tirupula et al., 2014).

The modulation of the activity of ERK1/2 by Ang-(1–7) has been analyzed previously. Several studies reported that this heptapeptide antagonizes the stimulation of ERK1/2 induced by various agents, such as bovine fetal serum (Gallagher and Tallant, 2004; Tallant et al., 2005), Ang II (Tallant and Clark, 2003; Su et al., 2006; Sampaio et al., 2007a), advanced glycated end products and TGF-β (Alzayadneh and Chappell, 2014). Additionally, some studies reported that Ang-(1–7) induced a direct activation of ERK1/2 (Zimpelmann and Burns, 2009; Liu et al., 2012). Participation of the MasR in these effects was suggested through the use of either antisense oligonucleotides for the receptor (Tallant et al., 2005), or the peptide D-Ala7-Ang-(1–7) (Su et al., 2006; Zimpelmann and Burns, 2009; Liu et al., 2012; Alzayadneh and Chappell, 2014). Our current observation that Ang-(1–7) caused an increase in the levels of phosphorylation of ERK1/2 both in cells that overexpressed the MasR and in control cells made it necessary to evaluate the endogenous expression of other receptors that could bind Ang-(1–7) in HEK293T cells. Because in control cells expression of AT2R was not detected and the endogenous expression of MasR was negligible in comparison with AT1R, it was possible that the latter was mediating the activation of ERK1/2 by Ang-(1–7). Lack of ERK1/2 activation induced by Ang-(1–7) in HEK293T cells whose AT1R expression was silenced indicated that this receptor was implicated in this response. On the other hand, the absence of response to Ang-(1–7) observed in HEK293T cells overexpressing MasR whose AT1R expression was silenced indicated that MasR alone was not involved in increasing phosphorylated ERK levels. Altogether, these results indicate that the attenuation of the response observed by overexpressing the MasR both in the concentration-response and in time-response analysis are a consequence of MasR ability to attenuate ERK activation induced by Ang-(1–7) over the AT1R. In this sense, it has been reported that the MasR acts as a physiological antagonist of the AT1R since it was observed that the coexpression of both receptors in CHO-K1 cells significantly decreased both the Ang II-induced production of IP1 and the increase of intracellular Ca^2+^ levels mediated by the AT1R (Kostenis et al., 2005). The mechanism behind this modulation could be the formation of MasR-AT1R heterodimers (Kostenis et al., 2005).

The analysis of the interaction between Ang-(1–7) and the AT1R originated discrepancies. Angiotensin-(1–7) has been found to compete with Ang II with either high (Gironacci et al., 1999) or low affinity (Rowe et al., 1995; Clark et al., 2001), while lack of competition was also reported (Bosnyak et al., 2011). Recently, Galandrin et al. (2016) by performing direct competition binding studies on membranes from AT1R–expressing HEK293T cells reported that Ang-(1–7) displaced Ang II with a Ki of 360 nM. In addition, Teixeira et al. (2017) by performing competition binding assays using ^3^H-Ang II in HEK293T cells transfected with AT1R reported that Ang-(1–7) binds to the AT1R yielding an affinity of ∼200 nM. We performed ERK1/2 phosphorylation assays in HEK293T cells endogenously expressing AT1R and barely detectable MasR levels using wild type cells and in conditions where MasR levels were very high. On the contrary, binding experiments in HEK293T cells were performed after overexpressing AT1R. The reasons for the differences between bindings results and the apparent high affinity of Ang-(1–7) in our results of ERK phosphorylation assays could be due to different relative expression levels of receptors that belong to the RAS (MasR, AT1R) that may influence Ang-(1–7) binding affinity given the pluridimensional action of Ang-(1–7) on different RAS receptors (Galandrin et al., 2016; Teixeira et al., 2017). Although the use of a system overexpressing a receptor is a useful tool to examine direct interactions with ligands, this may not be representative of the function of the receptor in its natural environment. In agreement with our results, Gaidarov et al. (2018) reported that in rat aortic endothelial cells (RAEC), a common source of endogenous rat angiotensin receptors, Ang-(1–7) inhibited the more potent, picomolar phase of Ang II-induced ERK1/2 phosphorylation at concentrations much lower than values obtained in presence of recombinant AT1 receptors. These authors postulated that in RAEC, angiotensin receptors may be organized in higher order signaling complexes with other receptors and signaling molecules, i.e., signalosomes, which may have higher affinities for Ang-(1–7) and that this higher order organization may not be present in recombinant cells or may be disturbed (Gaidarov et al., 2018). Finally, since the renal AT1R found on proximal tubules has very high affinity for Ang II (in the pM range) it is possible that in HEK293T cells, AT1R affinity is shifted to the left for both Ang II and Ang-(1–7). In this sense, Garcia and Garvin (1994) reported that Ang-(1–7) has a biphasic effect on transport, increasing fluid and bicarbonate absorption in the rat proximal straight tubule at 10^-12^ M concentration, while at 10^-8^ M concentration, Ang-(1–7) was found to decrease fluid absorption. Of note, both effects were blocked by losartan.

In glomerular mesangial cells, Ang-(1–7) has been shown to induce ERK1/2 through a MasR-mediated mechanism with no apparent participation of either AT1R or AT2R (Liu et al., 2012). The discrepancy between this study and current results could be due to the type of cells employed. For example, AT2R mRNA has been detected in mesangial cells (Tetzner et al., 2016) while we could not detect AT2R expression in HEK293T cells. Thus, in mesangial cells, a likely MasR-AT2R heterodimerization (Patel and Hussain, 2018) could contribute to Ang-(1–7) stimulation of ERK via MasR. Finally, the different levels of receptors attained under physiological conditions (Liu et al., 2012) versus the MasR overexpressing conditions used in the current study should be considered as a potential contributing factor to observed differences.

**Figure 7:**
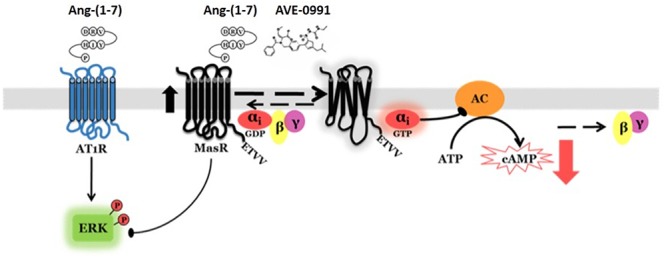
Proposed MasR signaling mechanisms. The MasR has constitutive activity toward reduction of cAMP levels. This effect is mediated by receptor coupling to Gαi adenylyl cyclase signaling pathway. MasR putative ligands [Ang-(1–7) and AVE 0991] amplify MasR activity. In addition to modulation of cAMP, the MasR attenuates Ang-(1–7)-induced ERK1/2 phosphorylation mediated by AT1R.

In summary, using an *in vitro* system, we demonstrated that human MasR has constitutive activity toward reduction of cAMP levels. We provided evidence in support for participation of Gαi_2_ protein in this effect. Incubation with putative ligands, either natural [Ang-(1–7)], or synthetic (AVE 0991), amplified this activity associated with MasR. In addition to modulation of cAMP, the MasR was shown to attenuate Ang-(1–7) induced ERK1/2 phosphorylation mediated by AT1R. We also provided evidence for lack of MasR-associated modulation of Ca^2+^ levels by Ang-(1–7) (Figure 7). This information could prove useful to better describe MasR physiological role and development of therapeutic agents that could modulate its action.

## Author Contributions

VB, EE, MS, DQ, and MM performed the experiments and analyzed data. VB, NF, FM, CD, and FD participated in the experimental design, analyzed the data, and wrote the manuscript.

## Conflict of Interest Statement

The authors declare that the research was conducted in the absence of any commercial or financial relationships that could be construed as a potential conflict of interest.
